# New Benefits of Hydroxychloroquine in Pregnant Women with Systemic Lupus Erythematosus: A Retrospective Study in a Tertiary Centre

**DOI:** 10.1055/s-0040-1715140

**Published:** 2020-11-30

**Authors:** Rahana Abd Rahman, Kyaw Min Tun, Ixora Kamisan Atan, Mohd Shahrir Mohamed Said, Ruslinda Mustafar, Ani Amelia Zainuddin

**Affiliations:** 1 Department of Obstetrics and Gynaecology, Universiti Kebangsaan Malaysia Medical Centre, Kuala Lumpur, Malaysia; 2 Department of Medicine, Universiti Kebangsaan Malaysia Medical Centre, Kuala Lumpur, Malaysia

**Keywords:** hydroxychloroquine, systemic lupus erythematosus, pregnancy

## Abstract

**Objective** To determine pregnancy outcomes in women with systemic lupus erythematosus (SLE) who were treated with hydroxychloroquine in a tertiary center.

**Methods** A retrospective study involving pregnant women with SLE who had antenatal follow-up and delivery in between 1 January 2007 and 1 January 2017. All participants were retrospectively enrolled and categorized into two groups based on hydroxychloroquine treatment during pregnancy.

**Results** There were 82 pregnancies included with 47 (57.3%) in the hydroxychloroquine group and 35 (42.7%) in the non-hydroxychloroquine group. Amongst hydroxychloroquine users, there were significantly more pregnancies with musculoskeletal involvement ( *p* = 0.03), heavier mean neonatal birthweight ( *p* = 0.02), and prolonged duration of pregnancy ( *p* = 0.001). In non-hydroxychloroquine patients, there were significantly more recurrent miscarriages ( *p* = 0.003), incidence of hypertension ( *p* = 0.01) and gestational diabetes mellitus ( *p* = 0.01) and concurrent medical illness ( *p* = 0.005). Hydroxychloroquine use during pregnancy was protective against hypertension ( *p* = 0.001), and the gestational age at delivery had significant effect on the neonatal birthweight ( *p* = 0.001). However, duration of the disease had a significant negative effect on the neonatal birthweight ( *p* = 0.016).

**Conclusion** Hydroxychloroquine enhanced better neonatal outcomes and reduced adverse pregnancy outcomes and antenatal complications such as hypertension and diabetes.

## Introduction

Systemic lupus erythematosus (SLE) is a complex multisystem autoimmune disease, affecting women primarily in their reproductive age. It is one of the main causes of maternal mortality in women between 15 and 24 years old. 1 The incidence of SLE varies from as low as 2.35 (per 100.000 persons-year) to as high as 8.1 in Taiwan. 2 3 In Malaysia, there is lack of national data, but the incidence in tertiary centers in Kuala Lumpur and Sarawak were 424 over 17 months and 633 over 12 months, respectively. 4 5 Diagnosis is made based on clinical features combined with laboratory findings according to the Systemic Lupus International Collaborating Clinics (SLICC) and the American College of Rheumatology Criteria for Classification of SLE. 6 7

Systemic lupus erythematosus activities are suppressed with nonsteroidal anti-inflammatory drugs (NSAIDs), immunosuppressive medication, or short courses of corticosteroids. There are associated risks of multiple organs damage due to the disease itself, or the medications used, specifically corticosteroids. 8 The survival rate and quality of life have significantly improved due to the advancement in disease management, leading to increasing numbers of successful pregnancies. These pregnancies are high-risk due to the associated fetal and maternal morbidity and mortality such as preeclampsia, intrauterine fetal loss, preterm delivery, intrauterine growth restriction (IUGR) and low birthweight. 9

During pregnancy, there is a risk of relapse as demonstrated by several published papers. 10 11 This can occur antenatally, as well as several months following delivery. 12 Women with lupus nephritis have a higher risk of relapse during pregnancy with worsening renal function despite aggressive therapy. Additionally, active disease of < 6 months prior to pregnancy is known to be the strongest predictor in disease flare and associated with worse pregnancy outcomes. 13

The mainstay of treatment in SLE consists of corticosteroids, low dose aspirin, and disease modifying anti-rheumatic drugs (DMARDs) such as hydroxychloroquine (HCQ). Hydroxychloroquine is an antimalarial drug that possesses anti-inflammatory and immunomodulatory properties via different molecular pathways. 14 Among the beneficial clinical effects is reduction in cholesterol and glucose levels as well as anti-thrombosis. More importantly, it plays a central role in the management of SLE due to reduction in disease flare associated with its use. 15 16 Other benefits of HCQ are prolongation of pregnancy and reduction in the rate of fetal growth restriction without teratogenic effects or long-term morbidities to the offspring. 17 18

In view of the various benefits of HCQ, the present study aimed to demonstrate the effects of HCQ in pregnant women with SLE.

## Methods

We conducted a retrospective cohort study in a tertiary hospital that is a referral center for rheumatology from 1 January 2007 till 1 January 2017. Ethics approval was obtained from the hospital research and ethic committee (FF-2017–433).

### Patient Involvement

The study population included pregnant women with SLE who fulfilled the criteria based on the Systemic Lupus International Collaborating Clinics (SLICC) and the American College of Rheumatology Criteria for Classification of SLE. 6 7 All of these women had antenatal management and deliveries in the tertiary center. Those who had antenatal care and/or delivered elsewhere were excluded. Patients were classified based on their HCQ use during pregnancy.

The gestational age of the pregnancies was determined based on the 1 ^st^ trimester dating scan. The data collected included demographic data such as age, ethnicity and parity, clinical characteristics of women with SLE such as association with antiphospholipid antibody, disease duration, organ involvement, disease activity at conception, concurrent medical illness, drugs used prior to pregnancy and the pregnancy outcomes, which included antenatal complications, pregnancy duration, birthweight, neonatal Apgar score, neonatal intensive care unit (NICU) admission and mode of delivery.

Activity during pregnancy was recorded using the SLE Pregnancy Disease Activity Index (SLEPDAI). Disease flare was defined as per Systemic Lupus Erythematosus Disease Activity Index (SELENA-SLEDAI). 19 20 Remission of disease was considered as absence of symptoms, signs and abnormal serology with or without maintenance treatment and low dose steroids (5mg or less). 21 Miscarriage was defined as fetal loss < 24 weeks of gestation. Recurrent miscarriage is defined as ≥ 3 consecutive pregnancy losses before 24 weeks. 22 Gestational age was considered preterm if < 37 weeks of gestation and term if at ≥ 37 weeks. Intrauterine growth restriction was defined as fetal weight < 10 ^th^ percentile for gestational age. Apgar score < 7 after 5 minutes of birth was considered abnormal.

The diagnostic of antenatal complications such as gestational diabetes was made based on modified glucose tolerance test (MGTT) of ≥ 5.6 mmol/l for fasting and ≥ 7.8 mmol/l for 2 hours postprandial. 23 Hypertensive disease in pregnancy included gestational hypertension and preeclampsia with or without existing secondary hypertension. Preeclampsia was defined based on criteria by the International Society for the Study of Hypertension in Pregnancy (ISSHP). 24

The demographic data included mean maternal age, ethnicity and parity. The clinical characteristics of the study population were presence or absence of antiphospholipid antibodies, mean disease duration, system involvement, history of recurrent miscarriages, concurrent medical illness, steroids use pre and during pregnancy and immunosuppressive drugs use pre and during pregnancy. The main outcome measures were the pregnancy outcomes consisting of antenatal complications, particularly hypertensive disease in pregnancy and gestational diabetes mellitus, mean duration of pregnancy, rate of fetal loss at ≥ 24 weeks, gestational age at birth, mean birthweight, rate of intrauterine growth restriction, mode of delivery, neonatal Apgar score at 5 minutes and NICU admission.

### Statistics

Data analysis was performed using IBM SPSS Statistics for Windows, Version 23 (IBM Corp., Armonk, NY, USA). Categorical variables were reported as frequency in percentage, and quantitative variables as mean and standard deviation (SD). The pregnancy outcome was tested with univariate analysis using the chi-squared and the Fischer exact test. A *p-value* < 0.05 was considered statistically significant. Multivariate analysis was done using logistic regression to identify the efficacy of HCQ on maternal outcomes and neonatal complications.

## Results

A total of 92 pregnancies were screened, 10 were excluded, and the remaining 82 were categorized into 2 groups; HCQ ( *n* = 47) and non-HCQ user ( *n* = 35). Table 1 demonstrates that there were no significant differences in the mean age (32.03 versus 31.5, *p* = 0.82), ethnicity ( *p* = 0.99) and parity ( *p* = 0.12). The majority of patients were multiparous Malay women.

**Table 1:** Demographic data of women with systemic lupus erythematosus grouped by hydroxychloroquine usage ( *n* = 82)

	HCQ n = 47 (57.3%)	Non HCQ n = 35 (42.7%)	OR (95%CI) *p-value*
**Mean age ± SD (years old)**	**32.03 ± 3.92**	**31.50 ± 3.54**	** 0.82 a**
**Ethnicity, n (%)**			0.99 b
**Malay**	35 (74.5)	26 (74.3)	
**Chinese**	9 (19.1)	7 (20.0)	
**Indian**	3 (6.4)	2 (5.7)	
**Parity, n (%)**			0.47 (0.18–1.21)
**Primipara**	20 (42.6)	9 (25.7)	0.12 c
**Multipara**	27 (57.4)	26 (74.3)	

Abbreviations: CI, confidence interval; HCQ, hydroxychloroquine; OR, odds ratio; SD, standard deviation.

a Student’s *t* -test.

b Contingency coefficient, df = 2.

c Chi-square test.

Table 2 showed that most of the patients in both groups had SLE without antiphospholipid antibody and this was not statistically significant ( *p* = 0.11). The 2 groups were similar in mean disease duration (7.06 versus 6.57, *p* = 0.44) and use of immunosuppressive drugs prior to ( *p* = 0.46) and during pregnancy ( *p* = 0.26). There were significantly more patients in the HCQ group who had musculoskeletal involvement ( *p* = 0.03). In the non-HCQ group, the rate of recurrent miscarriages was significantly higher (0% vs 17.1%, *p* = 0.003) with concurrent medical illnesses such as secondary hypertension, chronic kidney disease, thyroid, heart disease and idiopathic thrombocytopenic purpura (14.9% versus 42.9%, *p* = 0.005). Patients in the HCQ group used significantly more steroids prior to pregnancy (95.7% versus 82.9%, *p* = 0.05), but during pregnancy both groups were similar ( *p* = 0.22). There was a significantly lower prevalence of hypertension (6.4% versus 42.9%, *p* = 0.01) and gestational diabetes (0% versus 14.3%, *p* = 0.01) in the HCQ group. In the HCQ-treated group, the majority of patients (85.1%) were treated for > 6 months prior to pregnancy (results not shown).

**Table 2:** Clinical characteristics of women with systemic lupus erythematosus grouped by hydroxychloroquine usage ( *n* = 82)

	HCQ n = 47	Non HCQ n = 35	OR (95%CI) *p-value*
**Presence of antiphospholipid antibody, n (%)**			0.11 a
**No**	36 (76.6)	21 (60.0)	
**Yes**	11 (23.4)	14 (40.0)	
**Mean disease duration ± SD (years)**	7.06 ± 4.24	6.57 ± 3.56	0.44 a
**System involvement, n (%)**			
**Musculoskeletal**	29 (61.7)	13 (37.1)	2.63 (1.06–6.51)
			0.03 c
**Lupus nephritis**	23 (48.9)	22 (62.9)	0.54 (0.22–1.33)
			0.18 c
**Hematology**	24 (51.1)	12 (34.3)	2.09 (0.85–5.18)
			0.11 c
**CNS**	0	3 (8.6)	0.91 (0.83–1.01)
			0.07 c
**Recurrent miscarriage, n (%)**	0	6 (17.1)	0.83 (0.71–0.96)
			0.003 c
**Concurrent medical illness, n (%)**	7 (14.9)	15 (42.9)	0.23 (0.08–0.66)
**Secondary hypertension**	2 (4.3)	6 (15.8)	0.005 c
**Chronic kidney disease**	0	4 (10.5)	
**Thyroid disease**	1 (2.1)	2 (5.3)	
**Heart disease**	1 (2.1)	5 (13.2)	
**ITP**	2 (4.3)	1 (2.6)	
**Steroids use pre pregnancy, n (%)**	45 (95.7)	29 (82.9)	0.05 b
**5–10 mg**	32 (71.1)	20 (68.9)	
**10–20 mg**	5 (11.1)	4 (13.9)	
**>** **20 mg**	8 (17.8)	5 (17.2)	
**Steroids use during pregnancy, n (%)**	45 (95.7)	31 (88.6)	0.22 b
**5–10 mg**	28 (62.2)	19 (61.3)	
**10–20 mg**	11 (24.4)	7 (22.6)	
**> 20 mg**	6 (13.6)	5 (16.1)	
**Immunosuppressive drugs prepregnancy, n(%)**	23(48.9)	20(57.1)	0.46 b
**Azathioprine**	16 (34.0)	13 (37.1)	
**Cyclosporin A**	7 (14.9)	2 (5.7)	
**Both**	0	4 (11.4)	
**Immunosuppressive drugs during pregnancy, n (%)**	25 (53.2)	23 (67.7)	0.26 b
**Azathioprine**	17 (36.2)	18 (51.4)	
**Cyclosporin A**	6 (12.8)	2 (5.7)	
**Both**	2 (4.3)	3 (8.6)	

Abbreviations: CI, confidence interval; CNS, central nervous system; HCQ, hydroxychloroquine; OR, odds ratio; SD, standard deviation; ITP, idiopathic thrombocytopenic purpura.

a Student's *t* -test.

b contingency coefficient, df = 2.

c Chi-square test, difference expressed in odds ratio (95% CI), ITP-idiopathic thrombcytopenic purpura.

The numbers of term live births and miscarriages were similar between the 2 groups ( Table 3 ). The mean duration of pregnancy was significantly longer in the HCQ group (36.74 versus 34.79, *p* = 0.001) leading to a significantly heavier mean birthweight (2.52 versus 2.13, *p* = 0.02). There were no significant differences in fetal loss after 24 completed weeks, rate of live birth and miscarriages, newborns' Apgar score, NICU admission, mode of delivery and indications for cesarean section between the two groups. The most frequent indication for cesarean section in the non-HCQ and HCQ groups was preeclampsia (33.3%) and fetal distress (41.2%), respectively. However, more preterm delivery was found in the non-HCQ group, but it was not statistically significant (23.4% versus 40.0%, *p* = 0.25). Furthermore, IUGR was less frequent in the HCQ group (16.7% versus 35.7%, *p* = 0.08).

**Table 3:** Pregnancy outcome of women with systemic lupus erythematosus grouped by hydroxychloroquine usage ( *n* = 82)

	HCQ n = 47	Non HCQ n = 35	OR (95% CI) p value
**Antenatal complication**			
**Hypertension in pregnancy**	3 (6.4)	15 (42.9)	0.09 (0.02–0.35)
			0.01 c
**Gestational diabetes mellitus**	0	5 (14.3)	0.86 (0.75–0.98)
			0.01 c
**Mean duration of pregnancy ± SD (week)**	36.74 ± 2.01	34.79 ± 4.21	0.001 a
**Fetal loss ≥ 24 weeks, n (%)**	2 (5.3)	2 (7.1)	0.72 (0.09–5.47)
			0.75 c
**Gestational age at birth, n (%)**			0.25 b
**Term livebirth (≥ 37 weeks)**	26 (55.3)	14 (40.0)	
**Preterm livebirth (< 37 weeks)**	11 (23.4)	14 (40.0)	
**Miscarriage (< 24 weeks)**	10 (21.3)	7 (20.0)	
**Mean birthweight (kg) ± SD (range)**	2.52 ± 0.56	2.13 ± 0.79	0.02 a
**Intrauterine growth restriction, n (%)**	6 (16.7)	10 (35.7)	0.36 (0.11–1.16)
			0.08 c
**Mode of delivery, n (%)**			
**Vaginal**	18 (47.9)	13 (43.3)	0.91 b
**Assisted vaginal**	3 (7.9)	2 (6.7)	
**Caesarean section**	17 (44.7)	15 (50.0)	
**Apgar score**			
**≥ 7 in 5 minutes**	34 (94.4)	22 (78.6)	0.22 (0.04–1.17)
**< 7 in 5 minutes**	2 (5.6)	6 (21.4)	0.06 c
**NICU admission**	4 (11.1)	5 (19.2)	0.53 (0.13–2.18)
			0.37 c
**Indication for LSCS**			0.53 b
**Preeclampsia**	2 (11.8)	5 (33.3)	
**Fetal Distress**	7 (5.9)	2 (13.3)	
**Worsening Lupus**	1 (5.9)	2 (13.3)	
**Failed IOL/ Poor Progress**	2 (11.8)	2 (13.3)	
**Maternal Request/ Refuse TOS**	4 (23.5)	1 (6.7)	
**Breech**	1 (5.9)	1 (6.7)	

Abbreviations: CI, confidence interval; HCQ, hydroxychloroquine; IOL, induction of labor; LSCS; lower segment caesarean section; NICU, neonatal intensive care unit; OR, odds ratio; SD, standard deviation; TOS, trial of scar.

a Student's *t* -test.

b Contingency coefficient, df = 2.

c Chi-squared test, difference expressed in odds ratio (95% CI).

Two multivariate logistic regression analyses for preeclampsia and neonatal birthweight were performed ( Table 4 ). We included usage of HCQ ( *p* = 0.001), maternal age ( *p* = 0.63), concurrent medical illness ( *p* = 0.58), immunosuppressive treatment ( *p* = 0.06), and lupus nephritis ( *p* = 0.58), which were risk factors for preeclampsia. The usage of HCQ was still statistically significant ( *p* = 0.001), with HCQ treatment in pregnancy being independently protective against preeclampsia. Associated factors such as usage of HCQ ( *p* = 0.67), maternal age ( *p* = 0.33), concurrent medical illness ( *p* = 0.21), immunosuppressive treatment ( *p* = 0.98), lupus nephritis ( *p* = 0.45), gestational age at delivery ( *p* = 0.001) and duration of SLE ( *p* = 0.016) were included for neonatal birthweight. The gestational age at delivery ( *p* = 0.001) was a significant associated factor for neonatal birthweight and, in contrast, duration of SLE ( *p* = 0.016) had a significant negative effect.

**Table 4:** Multivariate models assessing the association between hydroxychloroquine usage and preeclampsia (Model I) and neonatal birthweight (Model II) incorporating potential confounders as covariates

	Multivariate Model I		Multivariate Model II	
	aOR (95% CI)	*p-value*	aß (95% CI)	*p-value*
HCQ usage	0.08 (0.02–0.36)	0.001	0.002 (−0.45–0.45)	0.67
Maternal age (years old)	1.04 (0.88–1.23)	0.63	0.01 (−0.02–0.03)	0.33
Concurrent medical illness	0.65 (0.15–2.91)	0.58	−0.12 (−0.30–0.07)	0.21
Immunosuppressive treatment	4.48 (0.92–21.88)	0.06	0.002 (−0.17–0.17)	0.98
Lupus nephritis	1.53 (0.34–6.77)	0.58	0.07 (−0.11–0.24)	0.45
Gestational age at delivery (week)	–		0.19 (0.16–0.21)	0.001
Duration of SLE (years)	–		−0.03 (−0.05 to −0.01)	0.016

Abbreviations: CI, confidence interval; HCQ, hydroxychloroquine; OR, odds ratio; SD, standard deviation; SLE, systemic lupus erythematosus.

## Discussion

The present study demonstrated the effects of HCQ regarding the pregnancy outcome in a single tertiary center. It is also a form of evaluation on the quality of care for women with SLE during pregnancy. This 10-year data has shown that despite the recommendation to continue HCQ treatment throughout pregnancy, there were a considerable number of women who were not treated. The main reasons were worry regarding its use in pregnancy and gastrointestinal upset. This was despite the knowledge that the use of HCQ in SLE had been established to be safe and beneficial. Although long-term use at high dosage may be associated with retinopathy, this can be avoided by ensuring the use of the optimum lowest dosage. The main positive effect of continuing HCQ in pregnancy is reduction in the incidence of flares, which led to longer pregnancy duration resulting in higher birthweight. 11 25 Additionally, women with positive anti-Ro/La antibodies benefited from the 64.5% reduction in the recurrence of neonatal congenital heart block in those with previous affected pregnancies. 26

Obvious findings that were similar to other published data were that the use of HCQ was associated with reduction in recurrent miscarriages and prolongation of pregnancy, resulting in heavier neonatal birthweight. 25 27 The role of HCQ in recurrent miscarriages due to antiphospholipid antibodies in women with SLE has not been well established. Women with positive antiphospholipid antibodies have been shown to have increased risk of thrombosis due to disruption of annexin A5 binding, which is believed to lead to recurrent miscarriages. 28 In vitro and animal studies had demonstrated that HCQ administration was associated with reversal of this pathological process. 29 30 Likewise, published clinical studies showed similar results. 31

There were two new benefits of HCQ observed in our study population. Use of HCQ was associated with lower incidence of hypertensive disease and gestational diabetes. There is limited evidence on the role of HCQ in hypertension. Theoretically, both SLE and preeclampsia shared a similar pathophysiology. 32 In line with this, an in vitro model of preeclampsia had demonstrated that HCQ improved tumor necrosis factor-α (TNF-α)-induced endothelial dysfunction. 33 Additionally, in a mouse model of SLE, treatment with HCQ was associated with reduction in hypertension, improvement in endothelial dysfunction and organ damage. 34 Recently, Seo et al 35 have found a significantly lower incidence of preeclampsia in SLE women treated with HCQ during pregnancy. Similarly, Kroese et al 25 observed a nonsignificant lower incidence of preeclampsia and HELLP syndrome.

Regarding gestational diabetes, there is growing evidence on the positive impact of HCQ on the disease. In line with our findings, several small studies have demonstrated the hypoglycemic and antidiabetic effect of HCQ. 16 More interestingly, the addition of HCQ to the first line oral hypoglycemic drugs or insulin in poorly controlled diabetics led to the improvement of HbA1c, fasting and postprandial glucose and dosage of insulin. 36 37 However, these were small studies. Hence, larger and longer duration of research is needed to establish the beneficial effects.

The strength of the present study was the duration of review of 10 years. The limitations of our study were the small sample size, short duration of study period, incomplete information for some pregnancies and inability to evaluate disease activity prior and during pregnancy. Both physicians and obstetricians need to educate their patients regarding the importance of continuing HCQ to reduce maternal and perinatal complications.

## Conclusion

More studies will be required to focus on hypertensive disease in pregnancy and gestational diabetes mellitus. Hydroxychloroquine may be a potential drug to improve the clinical outcomes of early onset severe preeclampsia or prevention against preeclampsia in women at high risk. Likewise, it may also be an adjunct therapy to improve the blood glucose control in diabetic patients during pregnancy, particularly in those who develop side effects with metformin.
